# Supplementary material for: Knowledge and practice of facemask disposal among university students in Thailand: A new normal post the COVID-19 pandemic
Source: PLoS One. 2023 Apr 13;18(4):e0284492. doi: 10.1371/journal.pone.0284492 (PMC10101522; doi:10.1371/journal.pone.0284492)
Supplement: Attachment — Submitted filename: PONE-D-23-04502.comments.docx [file pone.0284492.s001.docx]

**Reviewer-1**

The authors present an interesting piece providing data and perspective on knowledge and perspective of mask disposal among university students. The data gathering and statistical analysis has been rigorously done, as well as the discussion part. Several actions might be done to improve the paper more:
1. Comprehensive proofreading by native English speakers with relevant backgrounds might improve the paper (if this has not been done).
2. If considered relevant, the authors could present more perspective of knowledge and practice among university students during the pandemic from the more or less same demographic (in this case, e.g., Southeast Asia). These following papers could be considered to be cited: Adli I et al (2022) http://dx.doi.org/10.1371/journal.pone.0262827 ; Lazarus G et al (2021) https://bmcmededuc.biomedcentral.com/articles/10.1186/s12909-021-02576-0
On a side note, I support the authors in disseminating the paper more, such as by panel discussion, webinar, etc., involving central and local policymakers, local academicians, youth representatives, etc.
Regards,
Reviewer

**Reviewer-2**

In the present manuscript, the authors performed an investigation on the knowledge and practice of facemask disposal among university students in Thailand. The study is related to COVID-waste management, hence, it is timely and of interests. They used SPSS 25 to analyze the data obtained by the survey conducted within the university. The study is well conducted, however, this reviewer has certain points to mention before its final consideration.
1). My major concern is that after a hilarious pandemic like COVID-19, if up to 70% of students are confused regarding the proper management of used facemasks, it is a serious matter of concern. My point is that why the authors did not include the questionnaires on the topic to find out the reasons behind such lacking awareness among the students. It is expected that the younger generation should be more aware of that. Add a note and additional data.
2). Line 299-300: The authors should mention that why "male have a greater level of understanding than female"
3). The authors should add a separate recommendation section to overcome the issues find out by this study.
4). The authors should include some more articles on discussion like https://doi.org/10.1016/j.scitotenv.2020.141652
5). The authors should add the possible waste management techniques related to face masks.
