# Supplementary material for: Knowledge and practice of facemask disposal among university students in Thailand: A new normal post the COVID-19 pandemic
Source: PLoS One. 2023 Apr 13;18(4):e0284492. doi: 10.1371/journal.pone.0284492 (PMC10101522; doi:10.1371/journal.pone.0284492)
Supplement: Attachment — Submitted filename: Response to reviewers.docx [file pone.0284492.s002.docx]

**Response to Reviewers**

**TO:**	Editors of PLOS ONE

**FROM:**	Narisara Kaewchutima, Nopadol Precha, Netnapa Duangkong, Anthika Jitbanjong, Ni Made Utami Dwipayanti, authors of PONE-D-23-04502

**TITLE:**	“Knowledge and practice of facemask disposal among university students in Thailand: A new normal post the COVID-19 pandemic”

**RE:**	Response to reviewer comments

**DATE:**	March 28, 2023

| **Comments** | **Response** |
| --- | --- |
| **Reviewer 1#** | |
| 1. Comprehensive proofreading by native English speakers with relevant backgrounds might improve the paper (if this has not been done). | The manuscript has been proven by native English speaker as attached certificate. |
| 2.If considered relevant, the authors could present more perspective of knowledge and practice among university students during the pandemic from the more or less same demographic (in this case, e.g., Southeast Asia). These following papers could be considered to be cited: Adli I et al (2022): <http://dx.doi.org/>10.1371/journal.pone.0262827  Lazarus G et al (2021): <https://bmcmededuc.biomedcentral.com>  /articles/10.1186/s12909-021-02576-0 | Thank you very much for the great suggestions and references for discussion. We added more references on the discussion part to show the important role of the students in controlling and preventing the diseases during pandemic.  **Page 25 Line: 374-378.** |
| 3. On a side note, I support the authors in disseminating the paper more, such as by panel discussion, webinar, etc., involving central and local policymakers, local academicians, youth representatives, etc. | Thank you very much for your kind suggestion. We have planned to share our findings to the university and local policymakers. Also, we had shared this study in the international symposium of waste management. |
| **Reviewer 2#** | |
| 1. My major concern is that after a hilarious pandemic like COVID-19, if up to 70% of students are confused regarding the proper management of used facemasks, it is a among the students. It is expected that the younger generation should be more aware of that. Add a note and additional data. | The authors appreciate this critical point made by reviewer #2. We agree that this is important to be addressed.  It is also an interesting issue for us about poor knowledge on facemask disposal among the students. In addition, we are afraid we cannot add the questionnaire and additional data on awareness in the original sample group. Therefore, we will disseminate the findings of this study to the university’s policymaker to initiate the educational programs of infectious waste management in order to increase the awareness and proper practice in the students as mentioned in the recommendation.  **Page 26 line: 401-405.**  Also, added a note of attitude issue as a limitation.  **Page 26 line: 386-389.** |
| 2. Line 299-300: The authors should mention that why "male have a greater level of understanding than female" | Thank you very much for your comment. According to your comment, it is the results from the previous study that we used in discussion. However, we added the reasons why male have the greater level of understanding than female for more perfect discussion.  **Page 22 Line: 302-305.** |
| 3. The authors should add a separate recommendation section to overcome the issues find out by this study. | Thank you very much, we have separated the recommendation into the new section.  **Page 26-27 Line: 399-416.** |
| 4. The authors should include some more articles on discussion like https://doi.org/10.1016/j.scitotenv.2020.141652 | Thank you very much for the useful reference. We added more reference about incineration of used facemask in the discussion.  **Page 23 Line: 335-339.** |
| 5. The authors should add the possible waste management techniques related to face masks. | Thank you very much for useful suggestions on the additional management techniques related to facemask. We added possible waste management techniques in the discussion.  **Page 23 Line: 333-335 and 337-339.** |
